# Dynamic Remodeling of Human Arteriovenous Fistula Wall Obtained From Magnetic Resonance Imaging During the First 6 Months After Creation

**DOI:** 10.1016/j.ekir.2022.05.016

**Published:** 2022-05-21

**Authors:** Yingnan Li, Yong He, Isabelle Falzon, Brayden Fairbourn, Spencer Tingey, Peter B. Imrey, Milena K. Radeva, Gerald J. Beck, Jennifer J. Gassman, Prabir Roy-Chaudhury, Scott A. Berceli, Alfred K. Cheung, Yan-Ting Shiu, Hemodialysis Fistula Maturation Study, H. Feldman, L. Dember, A. Farber, J. Kaufman, L. Stern, P. LeSage, C. Kivork, D. Soares, M. Malikova, M. Allon, C. Young, M. Taylor, L. Woodard, K. Mangadi, P. Roy-Chaudhury, R. Munda, T. Lee, R. Alloway, M. El-Khatib, T. Canaan, A. Pflum, L. Thieken, B. Campos-Naciff, T. Huber, S. Berceli, M. Jansen, G. McCaslin, Y. Trahan, M. Vazquez, W. Vongpatanasin, I. Davidson, C. Hwang, T. Lightfoot, C. Livingston, A. Valencia, B. Dolmatch, A. Fenves, N. Hawkins, A. Cheung, L. Kraiss, D. Kinikini, G. Treiman, D. Ihnat, M. Sarfati, I. Lavasani, M. Maloney, L. Schlotfeldt, J. Himmelfarb, C. Buchanan, C. Clark, C. Crawford, J. Hamlett, J. Kundzins, L. Manahan, J. Wise, G. Beck, J. Gassman, T. Greene, P. Imrey, L. Li, J. Alster, M. Li, J. MacKrell, M. Radeva, B. Weiss, K. Wiggins, C. Alpers, K. Hudkins, T. Wietecha, M. Robbin, H. Umphrey, L. Alexander, C. Abts, L. Belt, J. Vita, N. Hamburg, M. Duess, A. Levit, H. Higgins, S. Ke, O. Mandaci, C. Snell, J. Gravley, S. Behnken, R. Mortensen, G. Chertow, A. Besarab, K. Brayman, M. Diener-West, D. Harrison, L. Inker, T. Louis, W. McClellan, J. Rubin, J. Kusek, R. Star

**Affiliations:** 1 Division of Nephrology, Baodi Clinical College of Tianjin Medical University, Tianjin, China; 2 Division of Vascular Surgery and Endovascular Therapy, University of Florida, Gainesville, Florida, USA; 3 Division of Nephrology & Hypertension, University of Utah, Salt Lake City, Utah, USA; 4 Department of Quantitative Health Sciences, Cleveland Clinic, Cleveland, Ohio, USA; 5 Department of Medicine, Cleveland Clinic Lerner College of Medicine of Case Western Reserve University, Cleveland, Ohio, USA; 6 Division of Nephrology & Hypertension, University of North Carolina, Chapel Hill, North Carolina, USA; 7 Department of Medicine, WG (Bill) Hefner VAMC, Salisbury, North Carolina, USA; 8 Vascular Surgery Section, Malcom Randall VAMC, Gainesville, Florida, USA; 9 Veterans Affairs Salt Lake City Healthcare System, Salt Lake City, Utah, USA

**Keywords:** black-blood magnetic resonance imaging, hemodialysis arteriovenous fistula, longitudinal study, vascular wall thickness

## Introduction

The nonmaturation rate of arteriovenous fistulas (AVFs) is high.^1^ The direct connection of the AVF vein and artery creates a short path that bypasses the distal high-resistance vasculature, resulting in an immediate increase in the flow rate and venous pressure. With the endothelium-dependent flow dilation and increased distention by elevated intraluminal pressure for the vein, the AVF lumen diameter increases immediately, resulting in a thinner wall and increased hoop stress within the wall immediately after AVF creation. Thereafter, in response to increased intramural stress, veins gradually thicken. Nevertheless, quantitative data describing how much and how fast human AVF venous wall thickness increases over time are very limited in the literature. Whether AVF wall thickness is correlated with lumen area is also not known.

Currently, measurements of human AVF vein wall thickness have been largely limited to histologic analysis of stenotic veins obtained during interventions,^2^ rarely noninvasively *in vivo*. To the best of our knowledge, only 1 study measured AVF vein intima-media thickness over time by ultrasound and only in 6 patients from 1 center.^3^ In addition, human AVF arterial walls and their changes over time have not been investigated. In this study, we used noncontrast, high-resolution black-blood magnetic resonance imaging (MRI) to evaluate the wall thickness and area of the AVF vein and proximal artery, along with the vein lumen area, within 6 months after AVF creation. We hypothesize that during AVF development, the AVF wall thickens in response to lumen enlargement. The detailed methods are given in Supplementary Methods.

## Results

### AVF Venous Anatomical Parameters Change With Time

**Figure 1:**
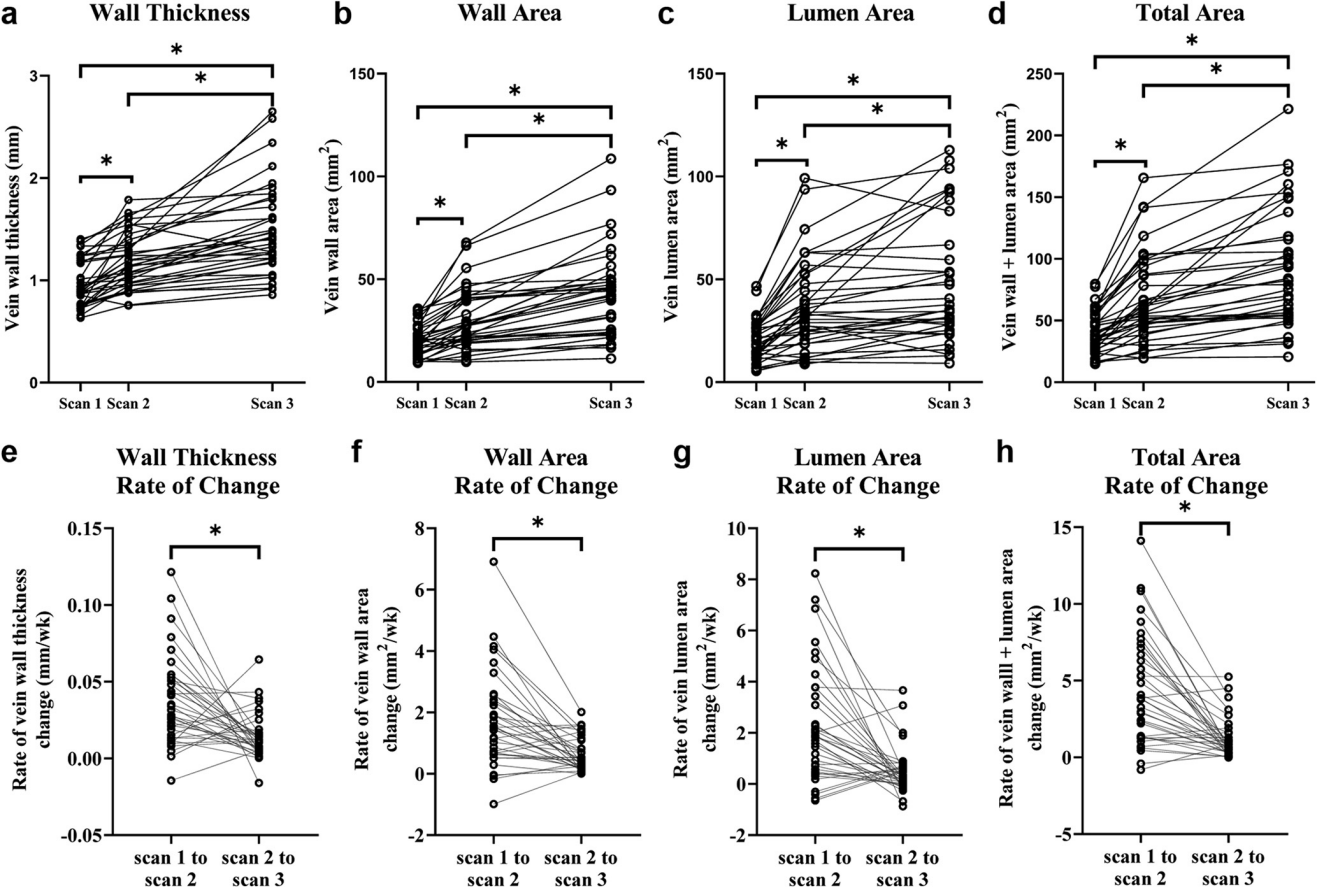
Venous parameters change with time. Top panels: Wall thickness (a), wall area (b), lumen area (c), and total (wall + lumen) (d) area at 3 scans. The distances between the scans were proportional to the time intervals between them. Bottom panels: The weekly rate of change between 2 scans for wall thickness (e), wall area (f), lumen area (g), and total (wall + lumen) area (h). All panels: The lines connect data points from the same patient. Scans 1, 2, and 3 were taken at 1 to 3 days, 6 weeks, and 6 months after fistula creation, respectively. ∗*P* < 0.05. *N =* 36.

Supplementary Figure S1a-h reveals the MR images and anatomical parameters of the AVF vein of 2 patients. These parameters vary along the vein length. The results presented in this study are based on parameters averaged over the entire AVF vein or artery for each patient. Supplementary Figure S2a-d displays the distributions of the 4 parameters (i.e., wall thickness, wall area, lumen area, total area) at each scan. From scan 1 to scan 2 and then to scan 3, most AVF veins had larger parameters as they adapted to the new hemodynamic conditions; and thus, the distribution curves shifted to the right. Figure 1a to d illustrates each patient’s parameters. Thus, most patients’ parameters increased with time (Supplementary Table S1). For all 4 parameters, the weekly changes in the early period (between scan 1 and scan 2) were significantly greater than those in the later period (between scan 2 and scan 3; Figure 1e–h; Supplementary Table S1).

### Associations Between AVF Venous Anatomical Parameters

**Figure 2:**
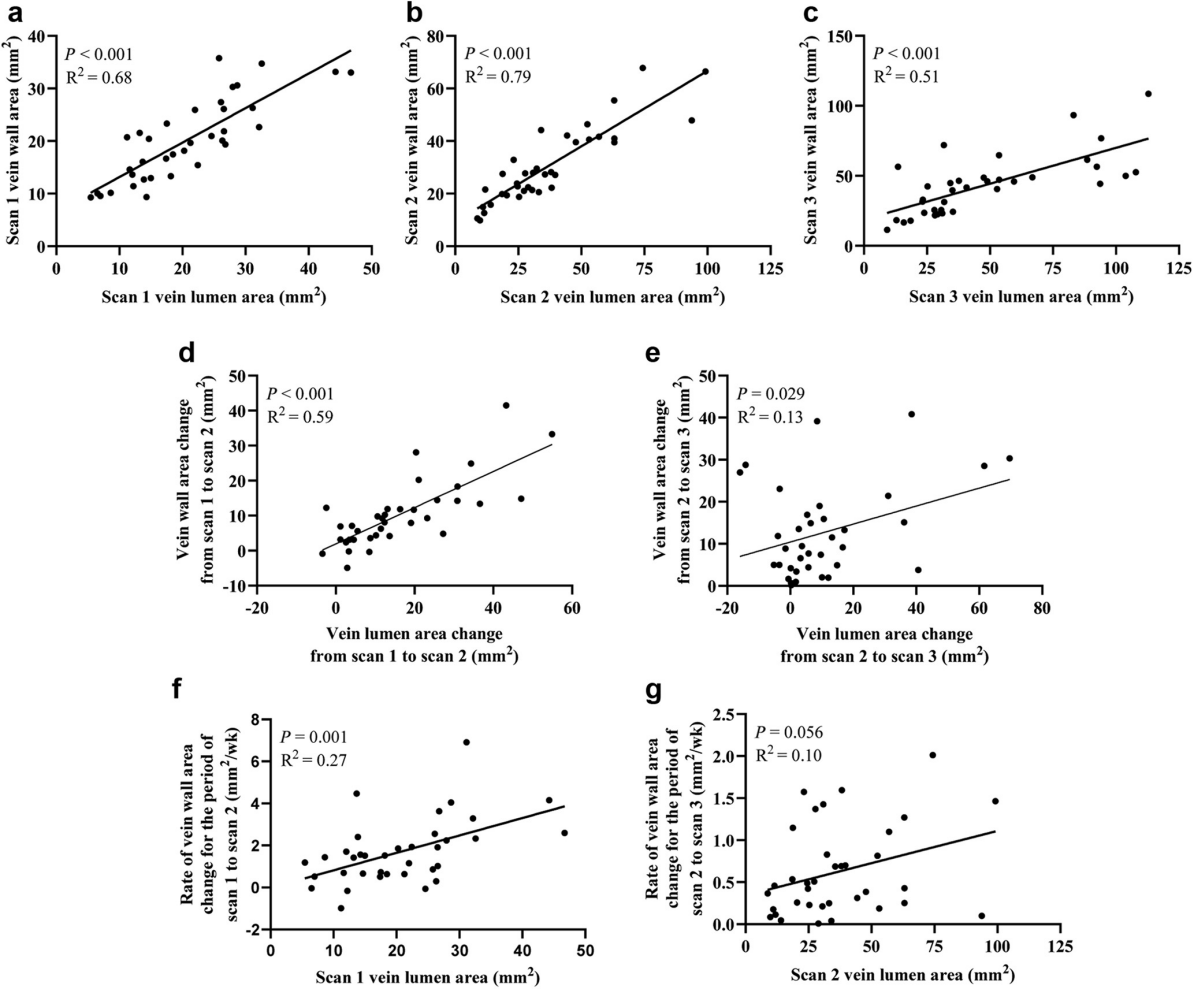
Association between venous parameters. The association between wall and lumen areas at (a) scans 1, (b) 2, and (c) 3. The association between wall area changes and lumen area changes in the early (d) and later (e) periods. The association between rate of wall area change from scan 1 to scan 2 and scan 1 lumen area (f). The association between rate of wall area change from scan 2 to scan 3 and scan 2 lumen area (g). The *P* values and R^2^ values are found in the plot for each panel, where the line is a linear trend line. Scans 1, 2, and 3 were taken at 1 to 3 days, 6 weeks, and 6 months after fistula creation, respectively. *N* = 36.

Venous wall area was highly correlated with lumen area at all 3 scans (the Pearson’s correlation coefficient, r = 0.82, 0.89, and 0.71 respectively; Figure 2a–c), as was wall thickness at scan 1 (r = 0.49, *P* = 0.003) and scan 2 (r = 0.50, *P* = 0.002), although less so and not statistically significantly at scan 3 (r = 0.14, *P* = 0.36). The wall area changes were also positively correlated with lumen area changes in both periods (r = 0.77, 0.36, respectively; Figure 2d–e), but wall thickness changes were not (*P* > 0.05). Therefore, as the vein lumen area expanded, the vein wall area also expanded, and a larger lumen expansion was accompanied by a larger wall area expansion. Nevertheless, we did not observe such a relationship between lumen expansion and wall thickness increase.

In each period, the weekly wall area change was positively correlated with the lumen area at the start of the period: r = 0.52 in the early (Fig. 2f) and r = 0.32 in the later periods (Fig. 2g). Thus, wall areas increased faster in veins with larger lumens, and this relationship was stronger in the early period. The weekly changes in wall thickness and area from scan 1 to scan 3 were also significantly positively correlated (r = 0.65; Supplementary Figure S3A).

### AVF Arterial Wall Anatomical Parameters Also Increase With Time and Are Associated With AVF Venous Wall Anatomical Parameters

Arterial parameters also increased with time (Supplementary Table S2). Thus, the distributions of AVF arterial wall thickness and area shifted right with successive scans (Supplementary Figure S4A and B). This pattern can be further observed from each patient’s data (Supplementary Figure S4C and D), with early weekly changes significantly greater than those in the later period (Supplementary Table S2 and Supplementary Figure S4E and F). Weekly changes in wall thickness and area from scan 1 to scan 3 were also significantly positively correlated (r = 0.73; Supplementary Figure S3B).

Venous and arterial wall areas were also significantly positively correlated at all 3 scans (Supplementary Figure S3C–E). Weekly venous and arterial wall area changes, however, were nonsignificantly correlated in the early (r = 0.20), later (r = 0.26), and overall (from scan 1 to scan 3, r = 0.36) observation periods.

### Comparison of Venous Parameters Between Maturated and Nonmaturated AVFs

Physiological maturation was defined as a venous flow rate ≥500 ml/min and a minimum venous lumen diameter ≥5 mm based on 6-week MRI scans as we previously described.^4^ Using this criterion, 24 AVFs maturated, and 12 did not. The maturated veins were significantly thicker than nonmaturated AVFs at scan 3 (1.62 ± 0.47 vs. 1.22 ± 0.25 mm, maturated vs. nonmaturated, *P* = 0.004; Supplementary Figure S5A and Supplementary Table S3). Other comparisons were not statistically different (Supplementary Figure S5B–H).

## Discussion

The arterial and venous walls in patients with end-stage kidney disease before fistula creation are thicker than in healthy patients due to medial hypertrophy and/or intima hyperplasia.^5^, ^6^, ^7^, ^8^, ^9^ From a single-center, ultrasound-based study of 6 patients in 3 months, the intima-media thickness of the AVF vein did not change, but the wall area increased from 1 week to 3 months.^3^ In the present multicenter, MRI-based study of 36 patients in 6 months, we found that the venous lumen area, wall area, and wall thickness all increased from 1 to 3 days to 6 months, more rapidly in the early than in the later periods. Importantly, venous wall and lumen areas and their rates of changes were positively associated, and the wall of the physiologically maturated vein was thicker than that of the nonmaturated vein at 6 months, suggesting that the AVF wall grows in conjunction with lumen enlargement, perhaps to maintain the structural strength and integrity of the wall. In addition, we found that the arterial wall area and thickness also increased from 1 to 3 days to 6 months, and there was a positive association between venous and arterial wall areas. Overall, our results suggest that the growth of venous and arterial walls may be needed for successful AVF maturation.

Our study is the first to use a noncontrast MRI method to longitudinally measure AVF wall area and thickness in patients with end-stage kidney disease. Several limitations exist. First, our approach cannot separate the intimal, medial, and adventitial layers in the wall. Second, it only included patients with high-quality scans at all 3 time points and without interventions. Third, we analyzed the AVF over 40 mm and averaged our findings; it is possible that different regions of the AVF (e.g., at vs. far away from anastomosis) may remodel differently. In conclusion, we have developed a reliable and reproducible protocol to use noncontrast MRI modality to measure wall area and thickness in patients with end-stage kidney disease. Therapies that promote the growth of both AVF lumen and wall may improve AVF maturation.

## Appendix

### Members of Hemodialysis Fistula Maturation Study

Chair, Steering Committee, University of Pennsylvania: H. Feldman; Clinical Centers, Boston University: L. Dember (principal investigator [PI]), A. Farber, J. Kaufman, L. Stern, P. LeSage, C. Kivork, D. Soares, M. Malikova; University of Alabama: M. Allon (PI), C. Young, M. Taylor, L. Woodard, K. Mangadi; University of Cincinnati: P. Roy-Chaudhury (PI), R. Munda, T. Lee, R. Alloway, M. El-Khatib, T. Canaan, A. Pflum, L. Thieken, B. Campos-Naciff; University of Florida: T. Huber (PI), S. Berceli, M. Jansen, G. McCaslin, Y. Trahan; University of Texas Southwestern: M. Vazquez (PI), W. Vongpatanasin, I. Davidson, C. Hwang, T. Lightfoot, C. Livingston, A. Valencia, B. Dolmatch, A. Fenves, N. Hawkins; University of Utah: A. Cheung (PI), L. Kraiss, D. Kinikini, G. Treiman, D. Ihnat, M. Sarfati, I. Lavasani, M. Maloney, L. Schlotfeldt; University of Washington: J. Himmelfarb (PI), C. Buchanan, C. Clark, C. Crawford, J. Hamlett, J. Kundzins, L. Manahan, J. Wise; Data Coordinating Center, Cleveland Clinic: G. Beck (PI), J. Gassman, T. Greene, P. Imrey, L. Li, J. Alster, M. Li, J. MacKrell, M. Radeva, B. Weiss, K. Wiggins; histology core facility, University of Washington: C. Alpers (PI), K. Hudkins, T. Wietecha; US core facility, University of Alabama at Birmingham: M. Robbin (PI), H. Umphrey, L. Alexander, C. Abts, L. Belt; vascular function core facility, Boston University: J. Vita (PI, deceased), N. Hamburg (PI). M. Duess, A. Levit; National Institute of Diabetes and Digestive and Kidney Diseases (NIDDK) biosample repository, Fisher BioServices: H. Higgins, S. Ke, O. Mandaci, C. Snell; NIDDK DNA repository, Fred Hutchinson Cancer Research Center: J. Gravley, S. Behnken, R. Mortensen; External Expert Panel: G. Chertow (Chair), A. Besarab, K. Brayman, M. Diener-West, D. Harrison, L. Inker, T. Louis, W. McClellan, J. Rubin; NIDDK: J. Kusek, R. Star.

## Supplementary Material

**Supplementary Methods.**

**Supplementary Results.**

**Supplementary References.**

**Figure S1.** Venous parameters along the fistula vein for selected patients.

**Figure S2.** Distribution of venous anatomical parameters.

**Figure S3.** Association between various parameters.

**Figure S4.** Distribution of arterial parameters and their changes with time.

**Figure S5.** Comparison of venous parameters between patients with maturated and nonmaturated arteriovenous fistulas.

**Figure S6.** Segmentation of the arteriovenous fistula lumen and wall.

**Table S1.** Mean arteriovenous fistula venous parameters (n = 36)

**Table S2.** Mean arteriovenous fistula arterial parameters (n = 36)

**Table S3.** Comparison of venous parameters between patients with maturated (n = 24) and not maturated (n = 12) arteriovenous fistulas.

**Table S4.** Baseline patient characteristics (n = 36)
