# Supplementary material for: Dynamic Remodeling of Human Arteriovenous Fistula Wall Obtained From Magnetic Resonance Imaging During the First 6 Months After Creation
Source: Kidney Int Rep. 2022 May 21;7(8):1905–9. doi: 10.1016/j.ekir.2022.05.016 (PMC9366358; doi:10.1016/j.ekir.2022.05.016)
Supplement: Supplementary File (PDF) [file mmc1.pdf]

Supplemental Material

Supplemental Methods

Supplemental Results

Figure S1. Venous parameters along the fistula vein for selected patients.

Figure S2. Distribution of venous anatomical parameters.

Figure S3. Association between various parameters.

Figure S4. Distribution of arterial parameters and their changes with time.

Figure S5. Comparison of venous parameters between patients with maturated and non-maturated arteriovenous fistulas (AVF).

Figure S6. Segmentation of the arteriovenous fistula (AVF) lumen and wall.

Table S1. Mean arteriovenous fistula (AVF) venous parameters (N=36)

Table S2. Mean arteriovenous fistula (AVF) arterial parameters (N=36)

Table S3. Comparison of venous parameters between patients with maturated (n=24) and not maturated (n=12) arteriovenous fistulas (AVF)

Table S4. Baseline patient characteristics (N=36)

Supplemental References

Supplemental Methods

Study patients and imaging

End-stage kidney disease (ESKD) patients undergoing new forearm or one-stage, upper-arm AVF placement were recruited at the hospitals of the University of Utah, University of Florida, and University of Cincinnati.[S1] Patients who were at least 18 years old, had a life expectancy of at least nine more months, and had no contraindications to MRI were recruited only after an AVF was actually created. The vein-end was manually sewn to the artery-side. The institutional review board in each university approved the protocols. All recruited patients provided written informed consent. Each patient underwent black-blood MRI scans at 1-3 days (scan_1), 6 weeks (scan_2), and 6 months (scan_3) post-AVF creation. The MRI methods and parameters were provided previously.[S1, S2] Scan_1 was performed to obtain post-surgery baseline values. Scan_2 was performed when patients underwent their standard-of-care ultrasound. Newly created AVFs are usually used for dialysis 6 months or later after creation, and thus scan_3 was performed at 6 months.

Three dimensional (3D) AVF reconstruction and parameters of measurement

Using the black-blood MR images, the lumen and total (lumen + wall) areas of the AVF vein were analyzed separately using Amira (Thermo Fisher Scientific, Waltham, MA; Supplemental Figure S6 a-d). The pixels belonging to lumen or total area were manually labeled to create a label field, which was then interpolated to obtain a slice interval of approximately 0.3 mm, achieving an equal spatial resolution in three dimensions. A surface representing the boundary between the lumen and wall, or between the wall and adjacent non-vascular tissue, was generated from the label field. Then, the surface was smoothed using the Vascular Modeling Toolkit (VMTK; vmtk.org). The centerline of the AVF venous lumen was extracted in VMTK and used to obtain the lumen or total area of the orthogonal cross sections at 1-mm intervals along the vein using a script written in Matlab (MathWorks, Inc., Natick, MA). The wall area was “the total area minus lumen area”. The wall thickness was “the total radius minus the lumen radius”, with the radii obtained from the corresponding areas assuming circular shapes. The wall’s area and thickness do not necessarily change in the same direction (i.e., increase or decrease at the same time) as the lumen size needs to be considered; thus, they were treated as independent variables in statistical analyses. The same procedure was performed on the proximal artery. A slight modification to the approach above, which was used for some patients, was to use the label field without interpolation and smoothing when the surface was generated in Amira and then use the centerline from VMTK to obtain the lumen or total area of the orthogonal cross sections in Amira. This modified approach generated values similar to those obtained from the method detailed above.

Inter-user agreement

The largest source of variation in these measurements (i.e., lumen area, wall area, wall thickness) between different users is likely manual segmentation, which is difficult to standardize. To examine inter-user variation, a random 10% (11) of the total scans (3×36 = 108) were segmented by two different users, with the parameters then extracted following identical procedures.

Statistical analysis

To avoid confounding by non-random missing data, we analyzed patients with all three MRI scans. A total of 36 patients who had adequate AVF wall images from all three scans and did not undergo surgical or endovascular interventions were analyzed and reported in this study (Supplemental Table S4). The other 16 patients who did not undergo surgical or endovascular interventions but had at least one in-adequate scan of the AVF wall were not analyzed. The number of subjects analyzed in all figures and tables are 36 unless otherwise specified. Data were presented as mean ± standard deviation or median (inter-quartile range), unless otherwise specified. Statistics were performed using GraphPad Prism (GraphPad Software, San Diego, CA). We compared measurements at consecutive scans using paired t-tests with Holm-Bonferroni multiple comparison adjustment. Paired t-tests were used to compare the two weekly rates of change, from scan_1 to scan_2 versus from scan_2 to scan_3. Simple linear regression was used to test for linear associations between parameters. Inter-user agreement was described by the concordance correlation coefficient (CCC, the R epiR package; www.r-project.org).[S3]

Differences were considered statistically significant if $P < 0.05$.

Supplemental Results

Inter-user agreement of MRI-derived wall area and wall thickness

The CCC using dual independent segmentations were 0.97 (95% confidence interval: 0.92-0.99) for vein and 0.91 (0.72-0.98) for artery wall areas, and 0.94 (0.81-0.98) and 0.78 (0.38-0.94) for vein and artery wall thicknesses, respectively. The mean absolute inter-user difference in artery wall thickness, the measurement with the lowest CCC, was only 0.07 mm, 6.6% of the mean artery wall thickness.

**Supplemental Table S1. Mean arteriovenous fistula (AVF) venous parameters (N=36)**			
Mean AVF venous morphological parameters			
	Day 1	Week 6	Month 6
Wall thickness (mm)	0.97 ± 0.23	1.19 ± 0.27	1.49 ± 0.45
Wall area (mm^2)	20.1 ± 7.8	30.5 ± 14.4	43.2 ± 21.5
Lumen area (mm^2)	20.6 ± 9.7	37.0 ± 22.4	47.8 ± 30.1
Total area (mm^2)	40.8 ± 16.7	67.4 ± 35.9	91.0 ± 47.9
Mean weekly rate of change in AVF venous morphological parameters			
	Day 1 – Week 6	Week 6 – Month 6	*P* value
Wall thickness (mm/week)	0.037 ± 0.030	0.014 ± 0.015	< 0.001
Wall area (mm^2/week)	1.70 ± 1.55	0.63 ± 0.54	< 0.0001
Lumen area (mm^2/week)	2.26 ± 2.26	0.47 ± 0.91	< 0.0001
Total area (mm^2/week)	4.42 ± 3.60	1.18 ± 1.28	< 0.0001

**Supplemental Table S2. Mean arteriovenous fistula (AVF) arterial parameters (N=36)**			
Mean AVF arterial morphological parameters			
	Day 1	Week 6	Month 6
Wall thickness (mm)	0.96 ± 0.16	1.08 ± 0.17	1.14 ± 0.19
Wall area (mm^2)	16.3 ± 5.7	20.1 ± 6.2	23.4 ± 9.7
Mean weekly rate of change in AVF arterial morphological parameters			
	Day 1 – Week 6	Week 6 – Month 6	*P* value
Wall thickness (mm/week)	0.020 ± 0.027	0.003 ± 0.005	0.001
Wall area (mm^2/week)	0.64 ± 0.78	0.15 ± 0.29	0.002

Data are presented as mean ± SD.

Supplemental Table S3. Comparison of venous parameters between patients with maturated (n=24) and not maturated (n=12) arteriovenous fistulas (AVF)

Mean AVF venous morphological parameters				
		Maturated	Not Maturated	*P* value
Wall thickness (mm)	Scan 1	1.04 ± 0.24	0.81 ± 0.09	0.27
	Scan 2	1.25 ± 0.28	1.09 ± 0.23	0.69
	Scan 3	1.62 ± 0.47	1.22 ±0.25	0.004
Wall area (mm^2)	Scan 1	21.4 ± 7.36	17.5 ± 8.14	0.98
	Scan 2	32.9 ± 13.8	25.8 ±15.2	0.78
	Scan 3	46.9 ± 18.5	35.8 ± 25.9	0.33
Lumen area (mm^2)	Scan 1	22.4 ± 9.96	17.1 ± 8.59	0.98
	Scan 2	40.7 ± 23.4	29.5 ± 19.0	0.72
	Scan 3	48.9 ±28.7	45.5 ± 33.9	0.99
Total area (mm^2)	Scan 1	43.9 ± 16.6	34.6 ± 15.8	0.98
	Scan 2	73.5 ± 36.1	55.3 ± 33.7	0.70
	Scan 3	95.8 ± 42.5	81.3 ± 58.0	0.86
Mean weekly rate of change in AVF venous morphological parameters				
		Maturated	Not Maturated	*P* value
Wall thickness (mm/week)	Scan 1 to Scan 2	0.033 ± 0.030	0.045 ± 0.028	0.46
	Scan 2 to Scan 3	0.018 ± 0.016	0.006 ± 0.009	0.50
Wall area (mm^2/week)	Scan 1 to Scan 2	1.86 ± 1.36	1.38 ± 1.91	0.65
	Scan 2 to Scan 3	0.69 ± 0.50	0.50 ± 0.60	0.97
Lumen area (mm^2/week)	Scan 1 to Scan 2	2.99 ± 2.39	2.17 ± 2.03	0.55
	Scan 2 to Scan 3	0.43 ± 0.93	0.81 ± 1.01	0.93
Total area (mm^2/week)	Scan 1 to Scan 2	4.85 ± 3.55	3.55 ± 3.69	0.53
	Scan 2 to Scan 3	1.11 ± 1.19	1.31 ± 1.49	0.99

Supplemental Table S4. Baseline patient characteristics (N=36)	
Variable	Median (IQR) or No. (%)
Age, year	58.0 (38.6-64.8)
Male sex	28 (77.8%)
Race	
White/Caucasian	21 (58.3%)
Black, African American, African	13 (36.1%)
Other race	2 (5.6%)
On dialysis	20 (55.6%)
Diabetes	19 (52.8%)
Hypertension	34 (94.4%)
Coronary artery disease	12 (33.3%)
Congestive heart failure	7 (19.4%)
History of cerebrovascular disease	7 (19.4%)
Upper-arm AVF	23 (63.9%)

IQR: interquartile range

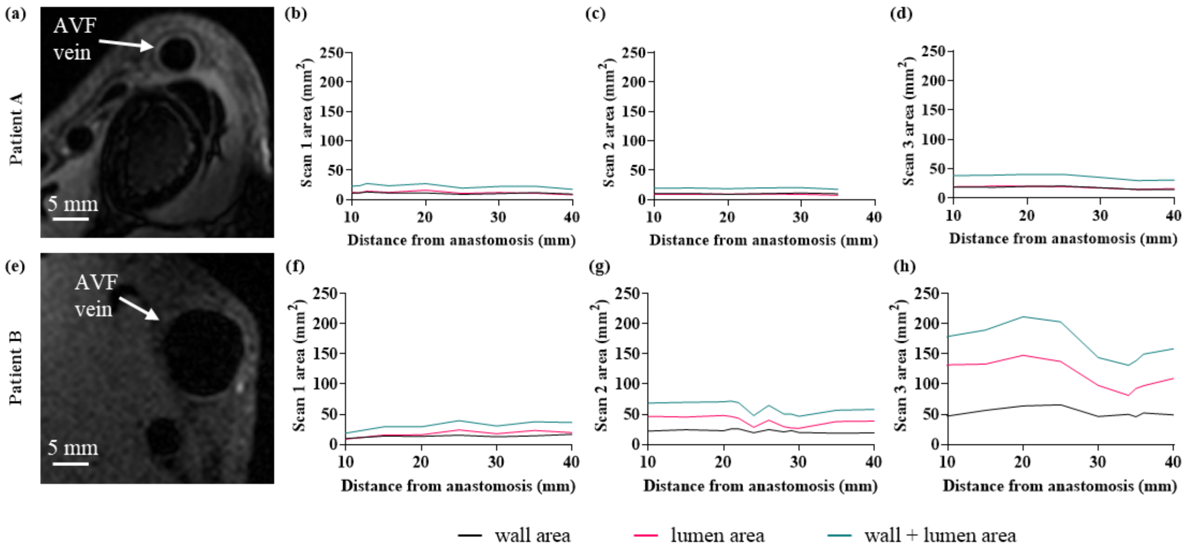

Supplemental Figure S1. Venous parameters along the fistula vein for selected patients. Magnetic resonance images at scan_3 (a, e) and the areas with respect to distance from the anastomosis (b-d, f-h) of two patients, Patient A (a-d) and patient B (e-h). Both patients had similar wall area and lumen area at scan_1. Patient B had a faster rate of change in wall area from scan_1 to scan_3 than patient A (1.58 versus 0.26 mm^2/week for B versus A). Similarly, patient B had a faster rate of change in lumen area from scan_1 to scan_3 than patient A (3.69 vs. 0.25 mm^2/week for B versus A).

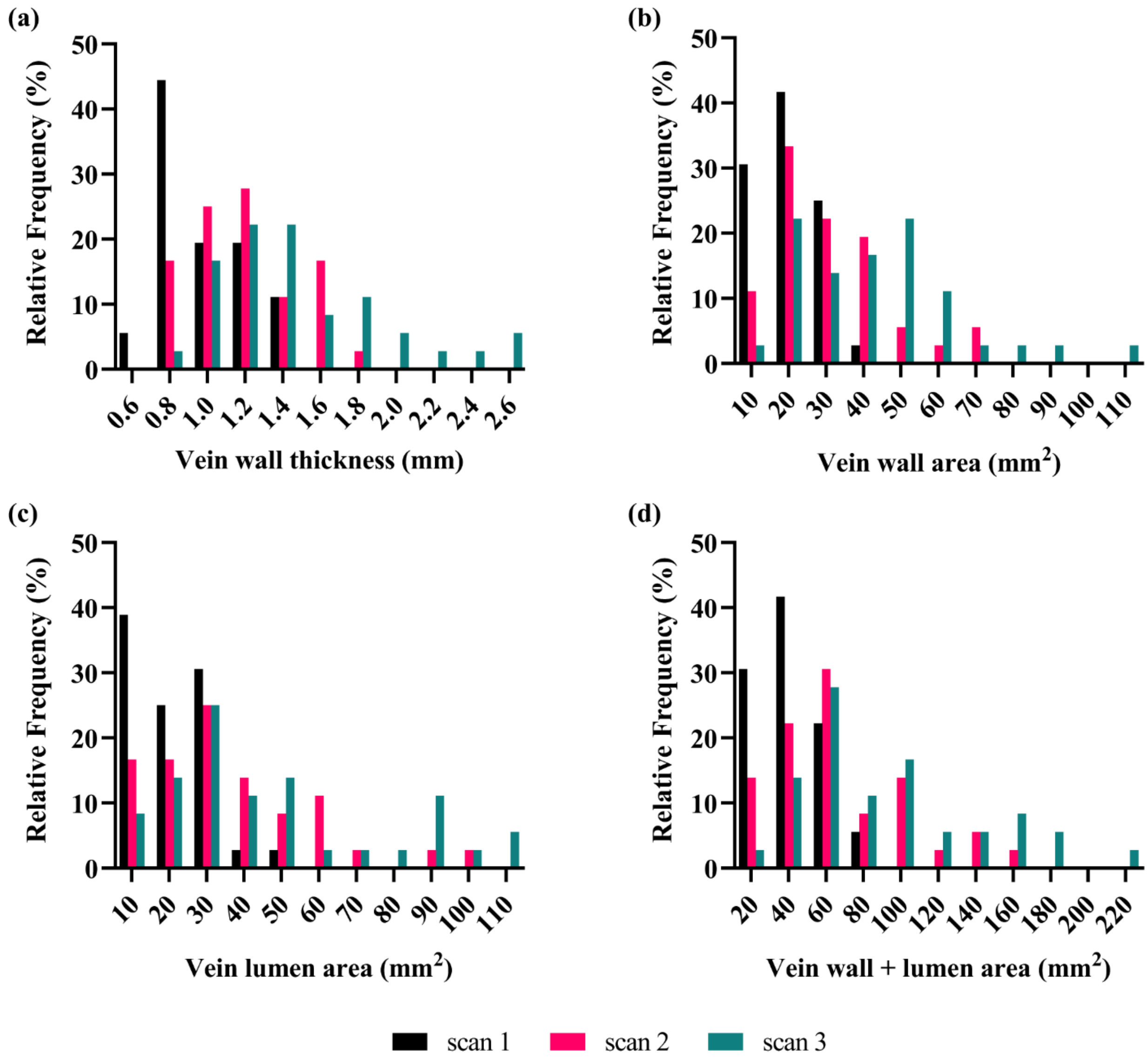

Supplemental Figure S2. Distribution of venous anatomical parameters. Venous (a) wall thickness, (b) wall area, (c) lumen area, and (d) total (wall + lumen) area at scans 1, 2, and 3 as indicated by the color code at the bottom. For all panels, area is referring to the cross-sectional area. The distance from the anastomosis to the slice where the last measurement was taken of all scans is 43.4 ± 11.0 mm (mean ± SD). Scans 1, 2, and 3 were taken at 1-3 days, 6 weeks, and 6 months after fistula creation, respectively. N=36.

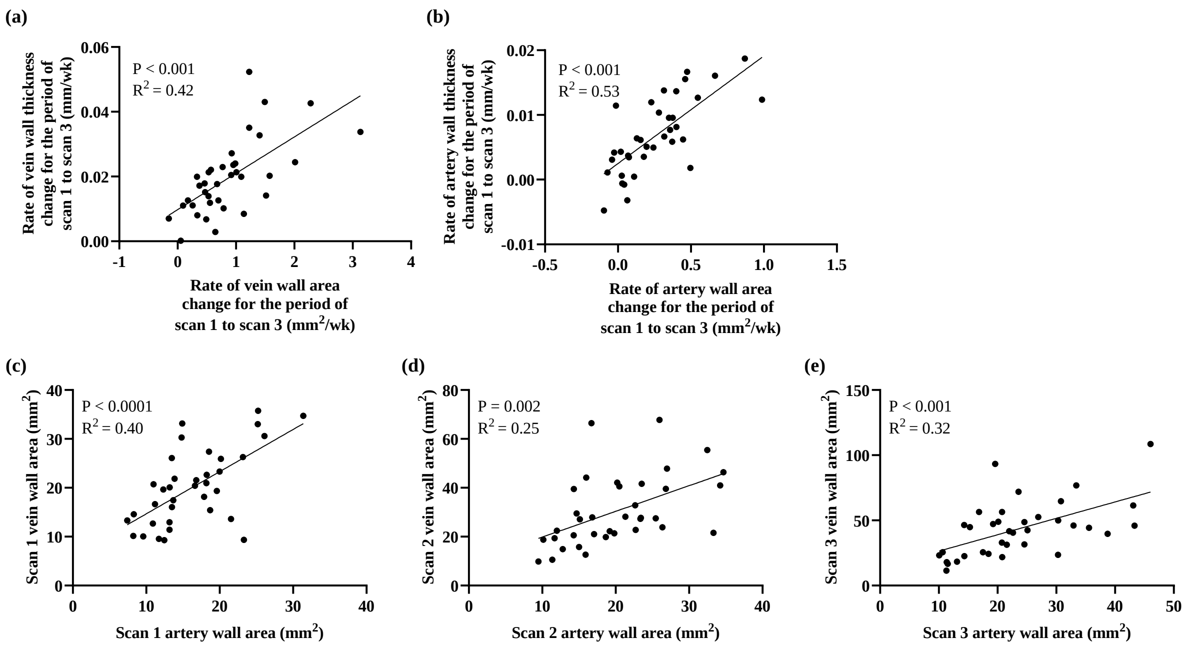

Supplemental Figure S3. Association between various parameters. Top panels: The association between the weekly wall thickness change and the weekly wall area change for the period of scan_1 to scan_3 for the (a) vein and (b) proximal artery. Bottom panels: The association between artery and vein wall area for (c) scans 1, (d) 2, and (e) 3. The P-values and R^2 values are shown on the plot for each panel, where the line is a linear trend line. Scans 1, 2, and 3 were taken at 1-3 days, 6 weeks, and 6 months after fistula creation, respectively. N=36.

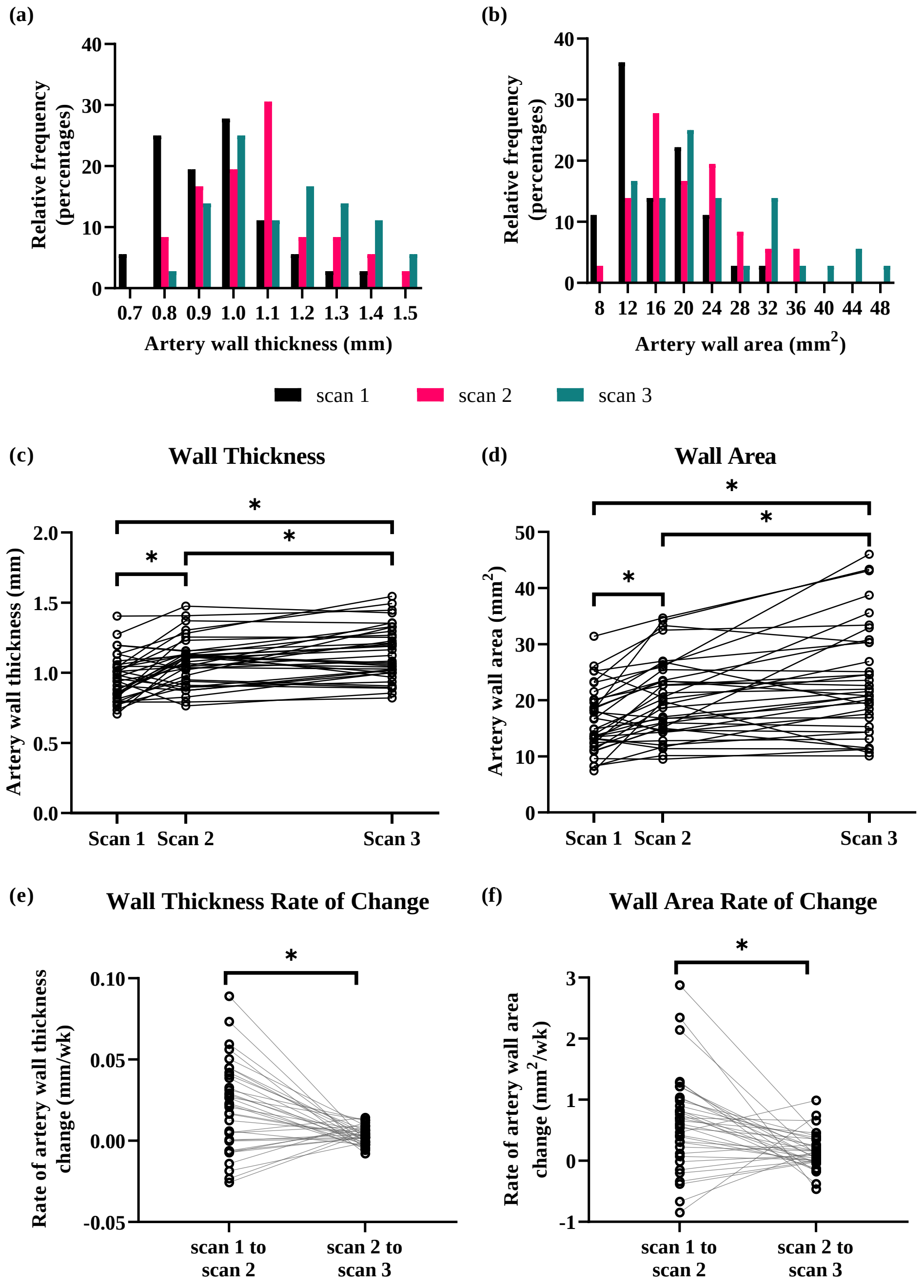

Supplemental Figure S4. Distribution of arterial parameters and their changes with time. Arterial (a) wall thickness and (b) wall area at scan_1, scan_2, and scan_3 as indicated by the color code at the bottom. Arterial wall thickness (c) and wall area (d) at the three scans and their weekly changes between two scans (e-f). The lines connect the points of the same patient. The distances between the scans were proportional to the time intervals between them. Note that area is referring to cross-sectional area. Scans 1, 2, and 3 were taken at 1-3 days, 6 weeks, and 6 months after fistula creation, respectively. * $P < 0.01$. N=36.

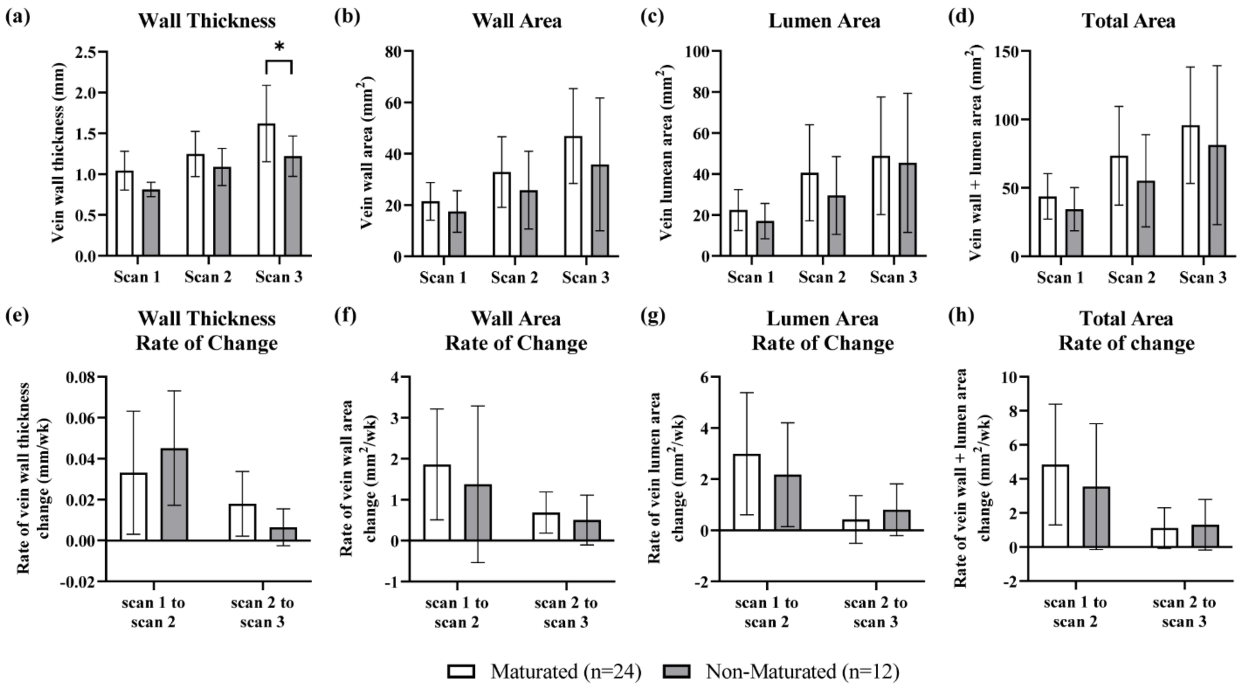

Supplemental Figure S5. Comparison of venous parameters between patients with maturated and non-maturated arteriovenous fistulas (AVF). Venous (a) wall thickness, (b) wall area, (c) lumen area, and (d) total (wall + lumen) area at scans 1, 2, and 3 for maturated and non-maturated AVFs. The weekly change between two scans for venous (e) wall thickness, (f) wall area, (g) lumen area, (h) total (wall + lumen) area for maturated and non-maturated AVFs. The error bars are ±SD. * $P = 0.004$.

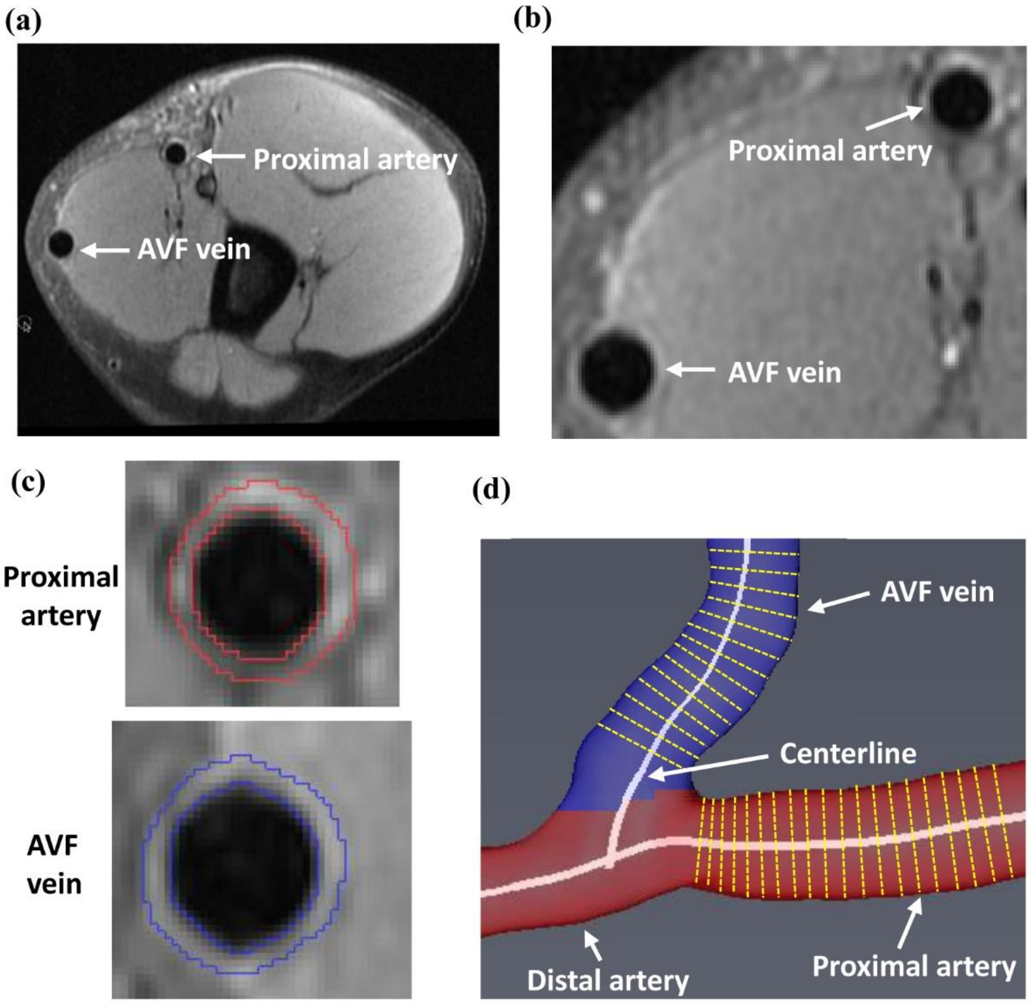

Supplemental Figure S6. Segmentation of the arteriovenous fistula (AVF) lumen and wall. (a) A slice of black-blood MRI taken at 6 weeks post AVF creation surgery. (b, c) Zoom-ins of black-blood MRI without (b) and with (c) segmentation of the lumen and wall of the AVF vein and proximal artery. (d) A zoom-in of the reconstructed AVF near the anastomosis. Yellow dashed lines indicate slices orthogonal to the centerline and spaced 1 mm apart. Of note, due to the 3-dimensionality, the intervals between lines may appear nonuniformly.

Supplemental References

S1. He Y, Northrup H, Roy-Chaudhury P, *et al.* Analyses of hemodialysis arteriovenous fistula geometric configuration and its associations with maturation and reintervention. *J Vasc Surg.* 2021; 73**:** 1778-1786.E1771.

S2. He Y, Shiu YT, Pike DB, *et al.* Comparison of hemodialysis arteriovenous fistula blood flow rates measured by Doppler ultrasound and phase-contrast magnetic resonance imaging. *J Vasc Surg.* 2018; 68**:** 1848-1857 e1842.

S3. Lin LI. A concordance correlation coefficient to evaluate reproducibility. *Biometrics.* 1989; 45**:** 255-268.
